# Human *LY6* gene family: potential tumor-associated antigens and biomarkers of prognosis in uterine corpus endometrial carcinoma

**DOI:** 10.18632/oncotarget.28409

**Published:** 2023-05-04

**Authors:** Luke A. Rathbun, Anthony M. Magliocco, Anil K. Bamezai

**Affiliations:** ^1^Department of Biology, Villanova University, Villanova, PA 19085, USA; ^2^Protean BioDiagnostics, Orlando, FL 32827, USA

**Keywords:** *LY6* gene family, uterine cancer, tumor-associated antigen, patient survival, biomarker

## Abstract

The human Lymphocyte antigen-6 (*LY6*) gene family has recently gained interest for its possible role in tumor progression. We have carried out *in silico* analyses of all known *LY6* gene expression and amplification in different cancers using TNMplot and cBioportal. We also have analyzed patient survival by Kaplan-Meier plotter after mining the TCGA database. We report that upregulated expression of many *LY6* genes is associated with poor survival in uterine corpus endometrial carcinoma (UCEC) cancer patients. Importantly, the expression of several *LY6* genes is elevated in UCEC when compared to the expression in normal uterine tissue. For example, *LY6K* expression is 8.25× higher in UCEC compared to normal uterine tissue, and this high expression is associated with poor survival with a hazard ratio of 2.42 (*p*-value = 0.0032). Therefore, some *LY6* gene products may serve as tumor-associated antigens in UCEC, biomarkers for UCEC detection, and possibly targets for directing UCEC patient therapy. Further analysis of tumor-specific expression of *LY6* gene family members and LY6-triggered signaling pathways is needed to uncover the function of *LY6* proteins and their ability to endow tumor survival and poor prognosis in UCEC patients.

## INTRODUCTION

Early detection and treatment of solid tumor malignancies has remained a major healthcare challenge. Identifying new tumor biomarkers, such as tumor associated antigens (TAAs), for diagnosis and as tumor targets for effective immunotherapies are critical needs. Antibody-based and cell-based immunotherapies (CAR-T cell therapy) targeting TAAs (e.g., CD19, Her-2, and CD52) have helped target blood cancers [1]. CD52 and Her-2 are both examples of TAAs that are often upregulated in breast cancer and can thus be targeted by antibody-drug conjugates, which help deliver cytotoxic drugs to cancer cells only [2]. Additional biomarkers on blood cancers and solid tumors will help expand this treatment repertoire to a variety of cancers arising from different tissues.

The *LY6* genes on chromosome 8q24.3 are of growing interest as this *LY6* locus is frequently amplified in human cancer [3]. Genes located at this locus include *LY6E, LY6L, LY6D, LY6K, LY6H, SLURP1, LYPD2, LYNX1, GML, and GPIHBP1*; these genes are syntenic to mouse chromosome 15. In total, the *LY6* gene family is comprised of at least 26 members, which are located on chromosomes 6, 11, and 19, in addition to chromosome 8 [4, 5]. Transcriptome analysis of pancreatic tumors has revealed upregulated expression of many *LY6* genes when compared to normal pancreatic tissue [3]. These findings are consistent with previous reports that show an increased expression of *PSCA* and other *LY6* genes (e.g., *SLURP1*) on a variety of neoplasms arising from prostate, bladder, ovarian, urothelial, and skin tissues [6–11]. Regardless of their chromosomal location, the *LY6* proteins are either glycosylphosphatidylinositol (GPI)-anchored to the membrane or are secreted [4]. A common feature present in all these proteins is the Ly-6/uPAR (LU) domain, which consists of 6–10 conserved cysteine residues [12]. These cysteine residues are arranged in specific spacing patterns that allow for disulfide bridge formation. The observed tertiary structure is a three-finger structural motif, which was first reported in the neurotoxin protein family [13]. Mouse *LY6* proteins expressed on immune and non-immune cells are reported to possess cell adhesion roles [14–21]. The functions of mouse Ly-6 orthologs in humans are observed in neuronal and other tissues where *LY6* proteins regulate nicotinic acetylcholine receptor [22]. Recent studies examining the function of *LY6* genes on human chromosome 8 have shown these genes to serve as biomarkers of poor cancer prognosis, and other studies have found them to be involved in cancer progression and immune escape [23, 24]. We report bioinformatic observations concerning upregulated expression and amplification of many *LY6* genes and their association with poor cancer patient survival in uterine corpus endometrial carcinoma (UCEC). Importantly, the expression of several *LY6* genes is elevated in UCEC when compared to the expression in normal uterine tissue.

## RESULTS

### Human *LY6* gene expression in normal and tumor tissues

*LY6* genes are expressed in a variety of normal, non-lymphoid tissues (Table 1). According to the GTEx Portal, tissues that normally express multiple *LY6* genes include the brain, esophagus, skin, and testis. In human tumors, expression of *LY6D*, *LY6E*, *LY6H*, and *LY6K* genes, which share the Ly-6/uPAR domain (Supplementary Figure 1), is significantly upregulated compared to normal tissues, and this is true for ovarian, colorectal, gastric, breast, lung, bladder, brain, cervical, esophageal, head and neck, and pancreatic tumors. For ovarian, colorectal, gastric, and breast cancers, this elevated expression is associated with poor overall patient survival [23].

**Table 1:** Human *LY6* Gene Family

*LY6* Member	UniProt ID	Chromosome	Cell surface (CS) or Secreted (S)	Normal tissue expression (From Gtex)	Function^*^
LY6E	Q16553	8	CS	Widely expressed. Highest in cervix, lung, ovary, liver, breast, and uterus (150–200 TPM)	Regulates T-cell proliferation, differentiation, and activation. May be involved in cancer metastasis. Possible modulator of nicotinic acetylcholine receptors. Main receptor for syncytin-A during placenta formation [41–45].
LY6L	H3BQJ8	8	CS	Highest in kidney, testis, and prostate (<1 TPM)	Function is inferred from homology. An important paralog for LY6L is LY6H.
LY6D	Q14210	8	CS	Highest in esophagus and skin (>1000 TPM), vagina (500 TPM)	Possible specification marker at earliest specification stage of lymphocytes between B- and T-cell development [46].
LY6K	Q17RY6	8	CS	Highest in testis, esophagus, and skin (<50 TPM)	Potential role in cell growth. Required for sperm migration into the oviduct and male fertility by controlling binding of sperm to zona pellucida [47, 48].
LY6H	O94772	8	CS	Primarily expressed in brain (>200 TPM)	Possible modulator of nicotinic acetylcholine receptors activity. Seems to inhibit alpha-7/CHRNA7 signaling in hippocampal neurons [41, 49].
SLURP1	P55000	8	S	Primarily expressed in esophagus and skin (>500 TPM), vagina (>100 TPM)	Displays antitumor activity. Late differentiation marker in skin. Possible modulator of nicotinic acetylcholine receptors. Possible regulator of intracellular Ca2+ signaling in T cells [50–56].
LYPD1	Q8N2G4	2	CS	Highest in brain (<50 TPM)	Possible modulator of nicotinic acetylcholine receptor activity [41, 49, 57].
LYPD2	Q6UXB3	8	CS	Highest in esophagus (>250 TPM), skin and vagina (<50 TPM)	No known or proposed function available.
LYPD3	O95274	19	CS	Highest in esophagus and skin (>1000 TPM), vagina (>500 TPM)	Supports cell migration. May be involved in tumor progression [58–61].
LYPD4	Q6UWN0	19	CS	Only in testis (>200 TPM)	No known or proposed function available.
LYPD5	Q6UWN5	19	CS	Highest in skin (<50 TPM), brain and esophagus (<10 TPM)	No known or proposed function available.
LYPD6	Q86Y78	2	Both	Highest in testis, brain, uterus, and bladder (<10 TPM)	Modulator of nicotinic acetylcholine receptor function in the brain [62].
LYPD6B	Q8NI32	2	CS	Highest in skin, testis, and stomach (<50 TPM)	Proposed modulator of nicotinic acetylcholine receptor activity [63].
LYPD8	Q6UX82	1	Both	Highest in colon and small intestine (<50 TPM)	Secreted form prevents invasion of Gram-negative bacteria in the inner mucus layer of colon epithelium [64].
LYPD9P	NA	1	NA	NA	Pseudogene
LYNX1	P0DP58	8	CS	Widely expressed. Highest in brain and heart (>50 TPM)	Interacts with nicotinic acetylcholine receptors [65].
CD59	P13987	11	Both	Widely expressed. Highest in lung and breast (>500 TPM)	Involved in signal transduction for T-cell activation complexed to a protein tyrosine kinase [46].
GML	Q99445	8	CS	Expressed in testis and adrenal gland (<5 TPM)	Possible role in apoptosis or cell-cycle regulation. Induced by p53 after DNA damage [66].
GPIHBP1	Q8IV16	8	CS	Widely expressed. Highest in breast and brain (>50 TPM)	Mediates transport of lipoprotein lipase from the basolateral to the apical surface of endothelial cells in capillaries [67–71].
LY6G5B	Q8NDX9	6	S	Widely expressed. Highest in brain, skin, ovary, cervix, uterus, and spleen (>50 TPM)	No known or proposed function available.
LY6G5C	Q5SRR4	6	S	Highest in testis and brain (<50 TPM)	Possible role in hematopoietic cell differentiation [72].
LY6G6C	O95867	6	CS	Highest in skin (>500 TPM)	No known or proposed function available.
LY6G6D	O95868	6	CS	Highest in testis and colon (<10 TPM)	Potential acetylcholine receptor inhibitor activity [46].
LY6G6F	Q5SQ64	6	CS	Highest in whole blood and testis (<10 TPM)	Potential role in downstream signal transduction pathways involving GRB2 and GRB7 [73].
PLAUR	Q03405	19	Isoform1: CS, Isoform2: S	Highest in whole blood and lung (>50 TPM)	Receptor for urokinase plasminogen activator and has role in localizing and promoting plasmin formation [74].
PSCA	O43653	8	CS	Highest in stomach (>1000 TPM)	Possibly involved in regulation of cell proliferation. Displays cell-proliferation inhibition activity *in vitro* [75, 76].

Abbreviation: NA: Not Available. Median expression is reported for each tissue. ^*^Some functions are proposed by similarity from mouse studies.

Analyzing *LY6* gene expression in a tumor may provide insight into a patient’s probability of survival or potential to respond to a certain therapy. Many *LY6* genes have recently garnered attention for their potential role as biomarkers of poor patient prognosis in pancreatic ductal adenocarcinoma [3]. *LY6K* is especially of interest as high mRNA expression of this gene is associated with poor patient survival in thyroid, kidney, uterine, and esophageal carcinomas [25]. Based on the results from these survival studies, we hypothesize that other *LY6* genes may also serve as biomarkers of poor prognosis in different cancers. To explore this, we analyzed RNA-seq data from The Cancer Genome Atlas (TCGA) for 20 different cancers, separated patients into high and low expression groups for each *LY6* gene, and compared overall survival between the two groups. We also compared *LY6* gene family expression in normal tissue to expression in tumor tissue to determine if *LY6* gene expression is upregulated in a given cancer. Highly upregulated *LY6* genes in cancer may allow for their detection. Additionally, *LY6* proteins on solid tumors may serve as targets for antibody and/or CAR-T cell therapies.

### Human *LY6* genes are biomarkers of poor prognosis and are upregulated in UCEC

**Figure 1:**
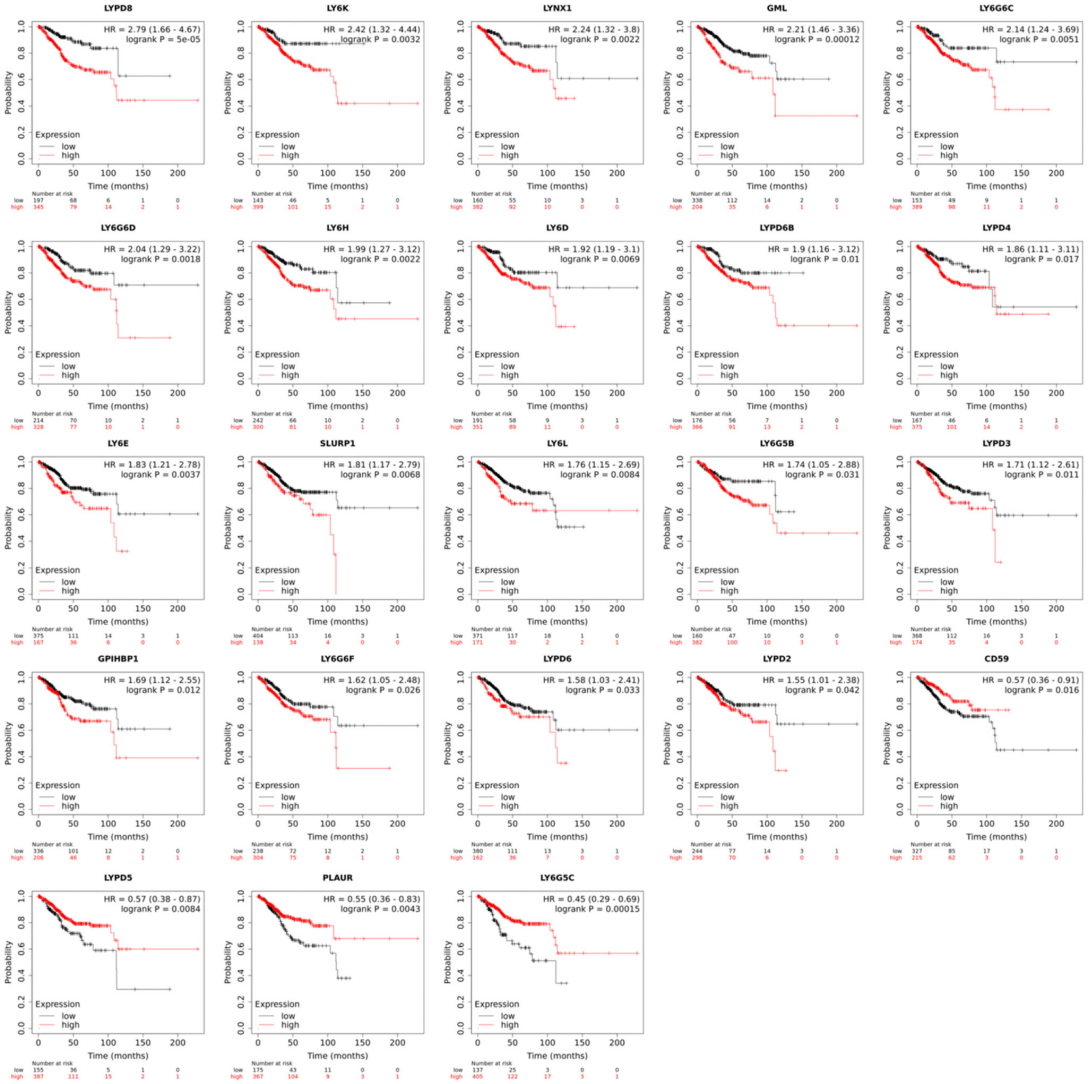
Overall patient survival in uterine corpus endometrial carcinoma (*n* = 543) based on high and low mRNA expression of a given human *LY6* gene. The red line represents the overall survival of patients with high expression of that gene, and the black line represents the overall survival of patients with low expression of that gene. A Cox proportional hazards model was used to determine if differences in survival between high and low expression groups were significant. RNA-seq data for UCEC was downloaded from TCGA, and overall survival was plotted using KM plotter.

Pan-cancer analysis of *LY6* gene expression revealed a negative correlation between mRNA expression and overall survival for most *LY6* genes in UCEC patients (Figure 1). In UCEC, there was a significant difference in survival between high and low expression groups for all *LY6* genes, except for *PSCA* and *LYPD1*. Of the 23 *LY6* genes for which there were significant differences in survival between high and low expression groups, 19 of these genes were associated with poor overall survival in high expression groups. *CD59*, *LYPD5*, *PLAUR*, and *LY6G5C* were all associated with increased survival in high expression groups. Negative and positive correlations between mRNA expression and overall survival were observed in other cancers as well. However, a focus was placed on UCEC since its *LY6* gene expression pattern was most similar to that of pancreatic ductal adenocarcinoma, which has already been described [3].

**Figure 2:**
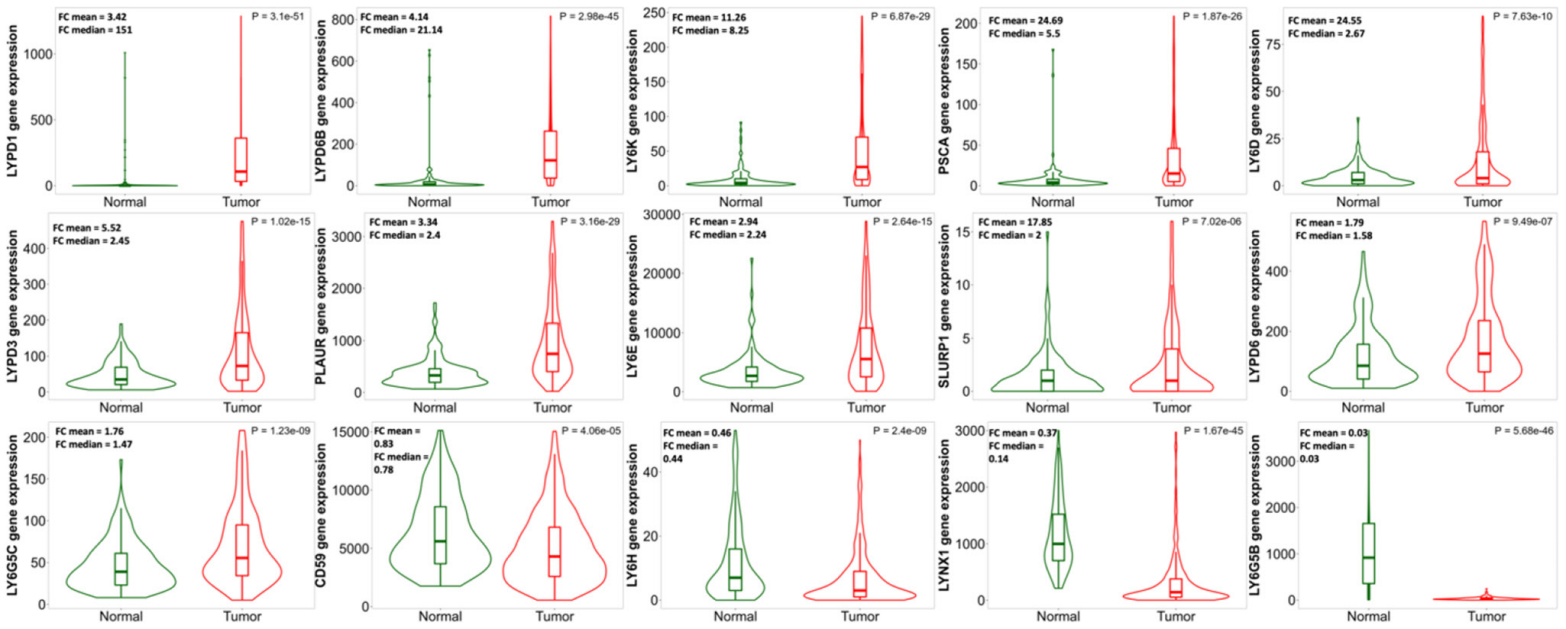
Comparison of human *LY6* gene expression in normal uterine tissue (*n* = 146) to expression in uterine corpus endometrial carcinoma (*n* = 547). Violin plots were generated using TNMplot, and fold changes in mean and median expression values were calculated for each gene. Many *LY6* genes show some degree of upregulation in UCEC with the exception of *CD59*, *LY6H*, *LYNX1*, and *LY6G5B*, which are downregulated. *LY6* genes not differentially expressed between normal and UCEC tissues are not shown.

Analysis of *LY6* gene expression in normal uterine tissue compared to UCEC revealed that mRNA expression of several *LY6* genes is upregulated in UCEC (Figure 2). mRNA expression of *LYPD1*, *LYPD6B*, *LY6K*, *PSCA*, *LY6D*, *LYPD3*, *PLAUR*, *LY6E*, *SLURP1*, *LYPD6*, and *LY6G5C* is significantly elevated in UCEC. mRNA expression of *CD59*, *LY6H*, *LYNX1* and *LY6G5B* is significantly reduced in UCEC. There is no significant change in mRNA expression for *LYPD8*, *LY6G6D*, *LYPD4*, *LY6L*, *LYPD2*, *LYPD5*, *LY6G6F*, *LYPD4*, *GPIHBP1*, and *GML*.

### *LY6* gene amplification in type I and II UCEC patients

The 8q24.3 locus contains several *LY6* genes and is frequently amplified in cancer with the reason not being understood. We observed increased amplification of this locus in UCEC compared to normal tissue. However, it was not previously known if 8q24.33 amplification is associated with more severe subsets of UCEC. To explore this, we separated UCEC patients based on their cancer type: uterine endometroid carcinoma (type I) and uterine serous carcinoma (type II) and compared amplification frequencies for each *LY6* gene. Type I tumors are estrogen driven and are associated with better prognosis whereas type II tumors are more aggressive and frequently carry genetic alterations in p53 and human epidermal growth factor-2 (HER-2) [26]. Of the UCEC patients represented in the TCGA pan cancer database, type I accounts for approximately 75% of cases, and type II accounts for approximately 20% of cases. A “mixed” type comprises the remaining 5% of cases [27, 28]. From our analyses, we found 8q24.3 amplification to be ~4× more prevalent in type II UCEC, and we also identified two additional *LY6*-containing loci that show increased amplification in this cancer type: 6p21.33 and 19q13.31 (Table 2).

**Table 2:** *LY6* gene amplification frequencies in type I and II UCEC patients

Gene	Locus	% Patients with gene amplification			*P*-value
		Uterine endometroid carcinoma (*n* = 394)	Uterine serous carcinoma (*n* = 108)	Total (*n* = 502)	
LY6L	8q24.3	0	0	0.00	>0.9999
LYPD1	2q21.2	0	0.93	0.20	0.2151
LYPD6B	2q23.2	0	0.93	0.20	0.2151
LYPD6	2q23.2	0	0.93	0.20	0.2151
CD59	11p13	0.25	0.93	0.40	0.3843
LYPD4	19q13.2	0	2.78	0.60	**0.0097**
LYPD3	19q13.31	0	3.70	0.80	**0.0020**
PLAUR	19q13.31	0	3.70	0.80	**0.0020**
LYPD5	19q13.31	0	3.70	0.80	**0.0020**
LY6G6D	6p21.33	0.76	3.70	1.39	**0.0416**
LY6G5C	6p21.33	0.76	3.70	1.39	**0.0416**
LY6G6C	6p21.33	0.76	3.70	1.39	**0.0416**
LY6G5B	6p21.33	0.76	3.70	1.39	**0.0416**
LY6G6F	6p21.33	0.76	3.70	1.39	**0.0416**
LYPD8	1q44	1.78	3.70	2.19	0.2616
GML	8q24.3	2.03	6.48	2.99	**0.0246**
LYPD2	8q24.3	2.03	6.48	2.99	**0.0246**
LYNX1	8q24.3	2.03	6.48	2.99	**0.0246**
LY6D	8q24.3	2.03	6.48	2.99	**0.0246**
LY6E	8q24.3	1.78	7.41	2.99	**0.0060**
PSCA	8q24.3	1.78	7.41	2.99	**0.0060**
SLURP1	8q24.3	2.03	6.48	2.99	**0.0246**
LY6K	8q24.3	2.03	7.41	3.19	**0.0099**
GPIHBP1	8q24.3	2.03	8.33	3.39	**0.0037**
LY6H	8q24.3	2.03	8.33	3.39	**0.0037**

*P*-values calculated by Fisher’s Exact test.

### *LY6* gene regulation in humans

Mouse Ly-6A/E protein expression is induced by type I (IFN-*α*/*β*) and type II (IFN-*γ*) interferons, which activate interferon regulatory factors (IRF) such as IRF9. IRFs activate expression by binding to cis-active interferon-sensitive response elements (ISRE) within distal enhancers of the mouse *Ly-6A/E* genes [29, 30]. To determine if human *LY6* gene family expression is regulated by type I IFNs and mediated by IRF9, ChIP-seq data were analyzed to identify IRF9 binding sites within distal enhancer elements of *LY6* genes. Bioinformatic tools were also used to infer ISRE-containing enhancers that possibly bind IRF9 and regulate *LY6* genes. The results from the ChIP-seq and GeneHancer data analyses are shown in Table 3. The publicly available ChIP-seq data from Qiagen and SPP did not return any IRF9 binding sites within the promoters or enhancers that regulate *LY6* gene family expression. However, GeneHancer was able to predict several distal enhancers that contain putative IRF9 binding sites and potentially regulate expression of the following *LY6* genes: *LY6E*, *LY6L*, *LYPD8*, *CD59*, *GPIHBP1*, *LY6G5B*, *LY6G5C*, *LY6G6C*, and *LY6G6D*.

**Table 3:** Human *LY6* gene regulation

*LY6* gene	Top TFs sites in gene promoter from Qiagen	Potential IRF9 binding site and distance from TSS (kb) from GeneHancer
LY6E	c-Rel, C/EBP*α*, En-1, IRF-1, LCR-F1, Lmo2, LUN-1, NF-*κ*B, NF-*κ*B1, TBP	Yes, +0.4
LY6L	NA	Yes, −62.3
LY6D	E2F, E2F-1, E2F-2, E2F-3a, E2F-4, E2f-5, HNF-4*α*1, HNF-4*α*2, LCR-F1, LUN-1	No
LY6K	c-Myc, FOXD1, FOXO4, GR, GR-*α*, GR-*β*, Max, USF-1	No
LY6H	HEN1, Olf-1, POU2F1, POU2F1a	No
SLURP1	AP-2*γ*, C/EBP*α*, GR, GR-*α*, GR-*β*, ITF-2, Nkx2-5, p53, Tal-1*β*	No
LYPD1	CUTL1, Evi-1, HTF, p53, SRF	No
LYPD2	AP-2*γ*, c-Fos, c-Jun, C/EBP*α*, GR, GR-*α*, GR-*β*, NF-*κ*B1, Nkx2-5, p53	No
LYPD3	AP-1, ATF-2, c-Jun, Sp1	No
LYPD4	GR, GR-*α*, GR-*β*, p53, PPAR-*α*	No
LYPD5	AP-1, ATF-2, c-Jun, HOXA5, LUN-1, Meis-1b, p53, POU2F1, POU2F1a, SEF-1	No
LYPD6	AML1a, Egr-2, GATA-3, POU2F1, POU2F1a, YY1	No
LYPD6B	AREB6, CUTL1, E2F-1, E47, FOXD3, FOXO3a, HOXA3, Pax-4a, Tal-1*β*, YY1	No
LYPD8	NA	Yes, −69.2
LYPD9P	NA	NA
LYNX1	E2F, E2F-1, E2F-2, E2F-5	No
CD59	GR	Yes, +14.2
GML	ER-*α*, Nkx3-1, Nkx3-1 v1/2/2/4, p53, Roaz	No
GPIHBP1	aMEF-2, C/EBP*α*, CHOP-10, GATA-2, Ik-3, Lmo2, MEF-2A, NF-1, NF-1/L, Pax-5	Yes, +6.5
LY6G5B	E47, Hand1, HNF-4*α*1, HNF-4*α*2, HTF, Pax-5, PPAR-*γ*1/2	Yes, +156.1, +67.3, +58.4, −761.7, −949.7
LY6G5C	AML1a, HSF2, LCR-F1, MRF-2, POU2F1a, PPAR-*γ*1/2, SRF, XBP-1	Yes, −53.4
LY6G6C	NF-*κ*B1, p53, Sp1	Yes, −104.5, −15.7
LY6G6D	C/EBP*α*, CHOP-10, ITF-2, MRF-2, NF-*κ*B1, PPAR-*γ*1, RFX1, Sp1, TaI-1*β*	Yes, +22.1, +110.9
LY6G6F	ITF-2, MRF-2, NF-*κ*B1, PPAR-*γ*1/2, RFX1, Sp1, TaI-1*β*	No
PLAUR	Sp1, STAT1, STAT3	No
PSCA	AML1a, AREB6, c-Ets-1, FOXJ2, GATA-1/2/3, RREB-1, ZID	No

Abbreviation: NA: Not Available.

## DISCUSSION

We carried out *in silico* analyses of all reported *LY6* genes, focused on their expression in different cancers, and analyzed patient survival by mining the TCGA database. We report that upregulated expression of many *LY6* genes is associated with poor cancer patient survival in uterine corpus endometrial carcinoma (UCEC). The overall survival data confirms that many upregulated human *LY6* gene products may serve as biomarkers for UCEC detection and may be useful in identifying high-risk UCEC patients. High expression of *LY6* proteins on the surface of tumor cells also makes them potential targets for cell-based and antibody-based immunotherapies. However, the magnitude of tumor expression above normal expression is critical to avoid autoimmunity and prevent targeting of self-tissues. Our mined transcriptomic information indicates that *LY6K* mRNA is significantly upregulated (>8 fold) in UCEC patient tumor tissues. If this mRNA is being translated to yield high levels of *LY6K* on the surface of uterine tumor cells, then cell-based therapies against *LY6K* might be able to selectively target and kill these cancer cells. In addition to these findings, we also report that a patient’s *LY6* gene amplification status may provide an alternative method for classifying type I and type II UCEC. Although rare, amplification of loci 8q24.3, 6p21.33, and 19q13.31 is more prevalent in type II UCEC and knowing a patient’s amplification status of these loci may help predict their likelihood of developing severe disease. Further analysis is needed, though, to determine if the *LY6* proteins encoded within these loci are involved in the development of the severe disease and poor outcomes associated with type II UCEC.

Transcriptional regulation of *LY6* genes is not well understood and has not been heavily investigated. Identifying the transcription factors that regulate *LY6* gene expression will help uncover the signaling pathways used by cancer cells and T cells to upregulate surface expression of *LY6* proteins. Expression of mouse *Ly-6A/E* is induced by type I (IFN-*α*/*β*) and type II (IFN-*γ*) interferons, but this has not been confirmed in human cell lines [29]. Interferon signaling is mediated by various IRFs, and many mouse *Ly-6* gene enhancers contain cis-active ISREs [30]. IRF9 is a transcription factor that is activated by type I IFN signaling and binds to ISREs within distal enhancer elements of interferon-stimulated genes (ISG) [31]. A majority of *LY6* genes contain regulatory sequences that can potentially bind IRF9 as well as an array of other transcription factors and activators (Table 3). The role of interferon responsive factors (e.g., IRF9), transcription factors, and other activators in upregulating the expression of *LY6* genes appears complex. Their interdependence, cause and effect relationship, or lack of, will require considerable experimental work including ChIP-seq and RT-qPCR analyses. Our *in-silico* analyses did not discover any common potential transacting factor binding sites within the *LY6* genes reported to be upregulated in UCEC patient tissues (Figure 2 and Table 3). Another future consideration is to understand the uterine tumor microenvironment, especially to delineate the expression of *LY6* proteins on the surface of tumor subpopulations and/or tumor-infiltrating lymphocytes. Multiplex immunohistochemistry would be a good technique to analyze the expression of *LY6* proteins on the surfaces of both cell types to assess their contributions to overall expression.

Further analysis of tumor-specific expression of the *LY6* gene family is needed to uncover the function of *LY6* proteins as well as the signaling pathways that these proteins trigger to endow tumor survival and poor prognosis in UCEC patients. A possible explanation, which would need to be tested, is that the ligands or receptors for *LY6* proteins are expressed in the uterine tumor microenvironment and drive tumor progression through binding that specific upregulated member of the *LY6* family. Further analysis of patient tumors is needed to uncover the functions of human *LY6* proteins as well as the signaling pathways that these proteins trigger to endow tumor survival and poor patient prognosis in other cancers as well. While upregulated expression of some *LY6* genes suggested poor patient prognosis, there are four *LY6* genes that showed the opposite, which was unexpected. High expression of *CD59*, *LYPD5*, *PLAUR*, and *LY6G5C* is associated with better patient outcome (Figure 1). Further analysis of patient tumors is needed to uncover the signaling pathways that these proteins trigger to endow beneficial UCEC patient prognosis.

## MATERIALS AND METHODS

### Analysis of human *LY6* gene expression and amplification in cancer

RNA-seq data for 20 different cancers were obtained from TCGA [27, 28]. Kaplan-Meier Plotter was used to separate patients into high and low expression groups for each *LY6* gene and then plot overall survival for each group [32, 33]. A proportional hazards model was used to calculate hazard ratios and *p*-values for each plot. GTEx Portal provided the top tissues in which *LY6* genes are normally expressed [34]. TNMplot was used to generate violin plots and compare *LY6* gene family expression in normal tissue to expression in tumor tissue [35]. Fold changes in expression were calculated using the median and mean expression values, and statistical significances were calculated using a Mann Whitney *U* test. cBioPortal was used to compare the amplification frequencies of *LY6* genes in type I and type II UCEC patients [36, 37]. Differences in amplification frequency were compared using a Fisher’s Exact test. The mixed UCEC group (*n* = 21) was not included in this analysis.

### Analysis of *LY6* gene regulation in humans

Top TFs in *LY6* gene promoters were provided by Qiagen and GeneCards [38]. Experimental ChIP-seq data for the *LY6* gene family were downloaded from The Signaling Pathways Project (SPP) and GeneHancer was used to predict distal enhancers that regulate human *LY6* genes [39, 40].

## SUPPLEMENTARY MATERIALS



## Abbreviations

*LY6*: Lymphocyte antigen-6
UCEC: uterine corpus endometrial carcinoma
TAA: tumor associated antigens
LU: Ly-6/uPAR domain
GPI: glycosylphosphatidyl-inositol
TCGA: The Cancer Genome Atlas
IFN: interferon
ISRE: interferon-sensitive response elements
ISG: interferon-stimulated gene
IRF: interferon regulatory factor
