# Supplementary material for: Human LY6 gene family: potential tumor-associated antigens and biomarkers of prognosis in uterine corpus endometrial carcinoma
Source: Oncotarget. 2023 May 4;14:426–37. doi: 10.18632/oncotarget.28409 (PMC10159366; doi:10.18632/oncotarget.28409)
Supplement: Supplementary file 1 [file oncotarget-14-28409-s001.pdf]

Human *LY6* gene family: potential tumor-associated antigens and biomarkers of prognosis in uterine corpus endometrial carcinoma

SUPPLEMENTARY MATERIALS

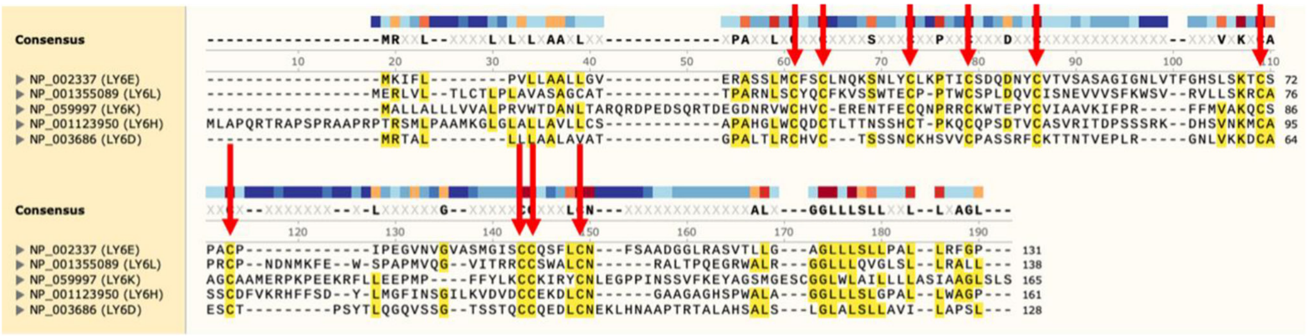

Supplementary Figure 1: Alignment of five human LY6 proteins generated in SnapGene. These five proteins were chosen due to their close proximity on Chr 8. The 10 conserved cysteine residues comprising the LU domain are indicated by the red arrows.
